# Standardized Patient Experience With the Fundoscopic Exam

**DOI:** 10.7759/cureus.83922

**Published:** 2025-05-11

**Authors:** Nisha Chadha, Joanne Hojsak, Neeti Parikh

**Affiliations:** 1 Ophthalmology, Icahn School of Medicine at Mount Sinai, New York, USA; 2 Pediatric Critical Care Medicine, Icahn School of Medicine at Mount Sinai, New York, USA; 3 Ophthalmology, University of California San Francisco, San Francisco, USA

**Keywords:** fundoscopy, medical student education, ophthalmology teaching, standardized patient, undergraduate clinical skills education

## Abstract

Introduction

Best practices for teaching physical examination skills to medical students often involve the use of standardized patients (SPs). However, few medical schools utilize SPs specifically for teaching fundoscopy, and limited information exists on how best to prepare SPs for this unique examination. This study aims to explore SPs’ attitudes, experiences, and comfort levels in teaching the fundoscopic exam.

Methods

Twenty-six SPs who participated in fundoscopy training sessions - 16 from the Icahn School of Medicine at Mount Sinai and 10 from the University of California, San Francisco - were invited to complete optional pre- and post-session surveys assessing their attitudes toward and comfort with the fundoscopic exam and pupil dilation.

Results

All 26 participants (100%) completed the pre-survey, and 21 (80%) completed the post-survey. Of these, 77% had worked as SPs for at least three years, and 73% had previous experience with fundoscopy sessions. During the training, 17 out of 21 SPs underwent pupil dilation, with four opting to dilate only one eye. Eighty percent reported satisfaction with the information provided about dilation, and 90% were satisfied with the guidance on what to expect from learners. The average reported comfort level during the exam was 3.76 out of 5. All SPs expressed willingness to participate in future fundoscopy sessions, with 90% open to doing so with dilation. Main concerns included the duration of dilation and extended exposure to bright light.

Conclusions

With proper orientation, SPs feel comfortable participating in fundoscopic examinations involving ocular dilation.

## Introduction

During the preclinical curriculum in medical school, students are taught various components of the physical examination. One common approach to teaching these skills involves the use of standardized patients (SPs). The concept of SPs was introduced in the 1960s by the late medical educator Dr. Howard Barrows, who later defined an SP as a simulated patient - a healthy individual trained to portray a patient’s illness in a consistent, standardized manner - as opposed to an actual patient trained to present their own condition in a standardized way [1].

SPs have become integral to medical education and are widely used as a consistent tool for teaching physical examination skills, history-taking, interview techniques, and the development of patient-physician communication skills [2-5]. Numerous studies have confirmed the educational value of SPs [6]. A recent review concluded that “students learned the most when SPs were used because they were able to teach students the skills that they needed in a safe learning environment… Students and residents reported increased confidence and clinical competence when performing new skills with SPs rather than with peer practice, virtual reality, or real patients in a clinical setting” [7].

Among the many clinical skills taught in medical school is the use of the direct ophthalmoscope. The ophthalmoscopic examination of the fundi is recommended as a core component of clinical skills curricula by the Association of American Medical Colleges [8]. However, proficiency with direct ophthalmoscopy is often challenging, and medical students frequently report low confidence in performing this exam [9,10]. Several studies have investigated different approaches to teaching fundoscopy, including simulation tools, 3D models, smartphone technology, the PanOptic ophthalmoscope, and peer-to-peer instruction [11-17]. Despite these innovations, to our knowledge, few, if any, studies have specifically examined the use of SPs in teaching fundoscopy.

While SPs are commonly used in teaching physical exam skills, their role in teaching fundoscopy remains underutilized. Fundoscopy presents unique challenges for SPs, including the need for pupil dilation and exposure to bright light. Currently, there is limited information on how best to prepare SPs for this specific examination. This study aims to evaluate SPs’ experiences, attitudes, and comfort levels with teaching the fundoscopic exam, particularly in the context of pupil dilation, through pre- and post-session surveys. The findings will help identify training needs and inform best practices for preparing SPs to support the teaching of this important clinical skill.

## Materials and methods

Clinical skills sessions focused on teaching fundoscopy to medical students were conducted at the University of California, San Francisco (UCSF) School of Medicine and the Icahn School of Medicine at Mount Sinai (ISMMS) during the fall of 2022 and spring of 2023. SPs, all of whom were paid actors, participated in these sessions. At both institutions, multiple sessions were held over the course of one to three days. Each session lasted 90 minutes and included small groups of four to eight students and one to two SPs. Students at ISMMS were trained using both the direct ophthalmoscope and the PanOptic ophthalmoscope, while students at UCSF used only the direct ophthalmoscope.

Prior to pupil dilation, the study authors met with each SP to review their ocular and eye examination history and to screen for potential risk factors for angle-closure glaucoma, including high hyperopia, a family history of angle-closure glaucoma, or any prior complications related to dilation. SPs were verbally informed about the dilation process, including potential side effects and risks. They were specifically advised of the risk of angle-closure glaucoma and the symptoms that should prompt them to seek immediate medical care.

All SPs who participated in the medical student fundoscopy teaching sessions at UCSF and ISMMS (total n = 26; 16 from ISMMS and 10 from UCSF) were invited to participate in the study using a total population sampling approach. Participation was voluntary, anonymous, and aligned with the principles of the Declaration of Helsinki. Informed consent was obtained, and participants voluntarily opted into the study. The pre-session survey was completed before the session began, and the post-session survey was completed within one week following participation.

The surveys were administered online, via Qualtrics XM at UCSF and REDCap at ISMMS, and were developed and reviewed by the study authors. While the instrument was not formally validated, survey items were carefully designed based on the authors' expertise and aligned with the study objectives. The surveys included a combination of multiple-choice, Likert-type, and short-answer questions. The pre-survey gathered information about participants’ general experience as SPs, their prior involvement in fundoscopy sessions, and their familiarity with pupil dilation. The post-survey assessed SPs’ perceptions of preparation, comfort, and overall experience during the session (see Appendix A and Appendix B for full survey instruments).

Only fully completed surveys were included in the analysis; incomplete responses were excluded. Quantitative data were analyzed using descriptive statistics. Qualitative analysis of free-text responses was conducted using basic inductive thematic analysis. Authors independently reviewed responses to identify recurring ideas and patterns, then met to compare interpretations and collaboratively group similar responses into overarching themes. Final themes were determined through discussion and consensus. The study was deemed exempt by the Institutional Review Boards at both UCSF and ISMMS.

## Results

A total of 26 SPs participated in this study, with 16 from ISMMS and 10 from UCSF. Participants ranged in age from 28 to 78 years, with a median age of 56. The gender distribution included 65% male (17/26), 31% female (8/26), and 4% identifying as other (1/26). All 26 SPs (100%) completed the pre-survey, and 21 SPs (80%) completed the post-survey.

The majority of participants (77%, 20/26) had worked as SPs for at least three years, and 73% (19/26) had previously participated in fundoscopy sessions in that role. Nearly all SPs (92%, 24/26) reported having undergone a general eye exam for their own health, with 79% (19/24) of those exams including pupil dilation.

During the teaching sessions, 81% (17/21) of SPs consented to have one or both pupils dilated, with 24% of those (4/17) choosing to have only one pupil dilated. Most SPs (80%, 16/20) reported being satisfied or very satisfied with the information provided about the dilation process. Similarly, 90% (19/21) expressed satisfaction with the guidance they received about what to expect from students during the fundoscopic exam.

Although fewer than one-third of SPs (6/21) were formally asked to provide feedback to students, nearly all (19/21) still offered informal feedback or suggestions. On a 5-point Likert scale (with 5 being “very comfortable”), the average comfort level reported during the exam was 3.76 (SD = 0.76). Notably, all SPs indicated a willingness to participate in future fundoscopy teaching sessions, with 90% (19/21) open to doing so even with pupil dilation.

Qualitative analysis of the pre-survey responses revealed three central themes: (1) concerns about the effects and duration of dilation; (2) personal interest in ocular health; and (3) a sense of responsibility in educating future physicians, particularly with regard to patient comfort (Table 1).

**Table 1:** Pre-survey themes

Pre-survey themes	Frequency	Percentage
Role in educating future physicians	15	41%
Interest in one’s own ocular health	9	24%
Effects and length of dilation	13	35%

Many SPs expressed concerns about pupil dilation and the duration of its effects. A common worry was the ability to travel home safely with blurred vision. One respondent noted, “I won’t be able to bike home after my pupils are dilated because my depth perception will be off.” Despite these concerns, most SPs felt well-informed about what to expect and were therefore prepared to manage the side effects. Additionally, many had participated in similar exam sessions in the past.

A notable motivation for participating was a personal interest in eye health. One SP shared, “I’m interested in all things eye health. My eyes have changed as I’ve gotten older, and I want to understand what’s going on.” Another added, “If they catch anything abnormal, they will relay that info!”

SPs also valued the opportunity to contribute a patient-centered perspective to the training of future physicians. Acknowledging the discomfort some patients may feel during the exam, SPs saw their role as essential in helping students learn to ensure patient comfort. As one participant stated, they hoped to help students “learn how to best utilize the instrument and attend to any discomfort concerns a patient may express.”

Qualitative analysis of the post-survey responses revealed three main themes: (1) discomfort with bright lights; (2) the importance of patient comfort during the exam; and (3) a recommendation for students to alternate eyes when performing the examination (Table 2).

**Table 2:** Post-survey themes

Post-survey themes	Frequency	Percentage
Students should alternate eyes being examined	5	24%
Consideration of patient comfort during the exam	7	33%
Discomfort with bright light	9	43%

Most SPs described the overall experience as “rewarding” or “positive.” However, many also reported discomfort and concern related to the duration of light exposure during the exam, noting that, as novices, “students spend a long time on each eye.” Some SPs expressed worry about the potential impact on their ocular health from prolonged exposure to bright light and felt more at ease when the instructor was an experienced clinician.

Concerns were also raised about the use of various devices and their differing light sources. One SP commented, “I don’t recall being told the students would use PanOptics as well…it wasn’t a problem, but I would have liked to know.” Another participant noted that students occasionally used “cell phone flashlights…with no hesitation, as if the two items were identical,” which raised concerns about eye safety. To reduce discomfort, several SPs recommended that students alternate eyes when performing examinations in succession.

Patient comfort emerged as a recurring theme in the post-survey responses, with SPs frequently emphasizing the importance of highlighting this aspect to students. Encouragingly, some SPs noted that students were attentive to their comfort during the exam. One SP recalled a student who was “very mindful of my comfort and verbalized reassurance and support.” The SP shared that they thanked the student and encouraged them to “keep doing it because it genuinely comforted me.”

## Discussion

SPs play a crucial role in modern medical education, and their perspectives and experiences can significantly inform efforts to retain and recruit SPs [18]. Talwalkar et al. emphasize this point in their paper on “creating an SP community,” offering 12 tips for running effective sessions with SPs. Recognizing the value of SPs’ experiences, particularly with sensitive physical exam techniques, this study aimed to explore their perspectives on teaching the fundoscopic exam [19].

Among this cohort of SPs, satisfaction with the overall experience and the preparatory information provided was high. Several key themes emerged that can guide future best practices for preparing SPs to teach students the fundoscopic exam.

The best practices identified in this study are outlined in Table 3. Although these practices are specific to this setting, they align with key domains of best practices proposed by the Association of Standardized Patient Educators, including safe work practices, respect, and preparation [20].

**Table 3:** Best practices for developing and preparing SPs for teaching students the fundoscopic exam SP, standardized patient

Best practice	Description
Prepare a standardized information session on dilation, side effects, and risks	Ensure participants are informed about the risk of angle-closure glaucoma, its associated symptoms, and assess for relevant risk factors. Inquire about ocular history and past eye exams.
Incorporate consideration of patient comfort into teaching exam technique	Consider alternating eyes and limiting the duration of the exam to maximize patient comfort.
Specify all devices students might be utilizing for ophthalmoscopy	List all potential devices, such as direct ophthalmoscopes, PanOptic ophthalmoscopes, and smartphone fundoscopy adapters.
Include an expert experienced in the fundus exam at the teaching session	Enlist an ophthalmology, optometry, or neurology expert (resident or faculty) to teach students the fundus exam.
Address concerns about transportation post-dilation	Consider and help develop transportation plans for participants, particularly regarding their ability to travel home independently after dilation.
Consider providing SPs with an informal report of their findings	Offer SPs an informal report of their findings, ideally provided by an expert. Alternatively, arrange for a formal eye examination following the session.

Firstly, considering concerns regarding dilation and its impact on visual function, a standardized information session on dilation, side effects, and risks is essential for the preparation and safety of these actors. All SPs were informed about the risk of angle-closure glaucoma, its associated symptoms, and the signs that should prompt them to seek medical attention. As outlined in the methods, the study authors met with all SPs prior to dilation, inquired about their ocular history and past eye exams, and assessed for risk factors related to angle-closure glaucoma, such as high hyperopia, a family history of angle-closure glaucoma, or previous problems with dilation. Furthermore, as an additional safety measure, we chose to use only tropicamide for dilation instead of both tropicamide and phenylephrine, given that the combined effect of the two drops may increase the risk of angle-closure glaucoma. Using only one drop instead of two helps reduce this risk. The information sessions should also specify all devices that students might use during ophthalmoscopy practice, such as direct ophthalmoscopes, PanOptic ophthalmoscopes, or smartphone fundoscopy adapters.

Secondly, post-survey themes were centered around patient comfort. The findings suggest that patient comfort can be optimized by incorporating key strategies, such as limiting light exposure by shortening the exam duration and alternating eyes between students during the exam.

Additionally, the survey data revealed that SPs felt more comfortable and secure when an expert led the session. This indicates that the most effective approach for conducting the session, benefiting both SPs and students, would be to have an ophthalmology, optometry, or neurology expert (such as a resident or faculty member) serve as the instructor.

Furthermore, due to concerns about the impact of dilation on their ability to travel home independently, transportation plans and support should be considered for SPs who are personally contributing to medical education.

Finally, given SPs’ interest in their ocular health, educators might consider offering SPs an informal report of their findings or arranging for a complimentary eye examination following the session.

While formal feedback was not explicitly evaluated in this study, most SPs mentioned that they provided some form of feedback or suggestions to students during the session. SPs play a critical role in medical education by offering valuable feedback to students. Although this study did not collect direct data on student learning outcomes or experiences, other studies have demonstrated that SPs can enhance students’ performance on examination skills and contribute to a more positive learning experience, thus increasing student satisfaction [21,22]. One potential direction for future practice could be to explore the impact of formal feedback training for SPs. This type of training could help build SPs’ confidence and support the development of more structured, effective feedback during student encounters [23]. However, further research is required to assess the relevance and feasibility of such training in this context.

While this study offers valuable insights into SP preferences for teaching fundoscopy, it has several limitations. The small sample size limits the generalizability of the findings. To increase generalizability, the study was conducted at two institutions. Additionally, there is a sampling bias, as most participants had prior eye exams, many of which included dilation, and were therefore likely familiar with what to expect. This experience may differ for SPs who have never undergone an eye exam. Including a larger number of SPs without previous eye exam experience could provide additional insights. The study also lacks a control or comparison group, such as SPs in non-fundoscopic exams, making it difficult to determine if the findings are unique to the fundoscopic setting or broadly applicable. Moreover, the study relied on a short-term assessment, with the post-survey completed within one week of the session. A follow-up survey to assess long-term attitudes or SP retention could be important for future recruitment and session planning. Additionally, understanding whether concerns raised by SPs are alleviated after attending multiple sessions could offer more insights into SP retention and the lasting effects of the experience.

## Conclusions

SPs can play an important role in teaching medical students the fundoscopic exam. This study provides valuable insights into best practices for preparing SPs for such sessions. With proper orientation, SPs are comfortable participating in fundoscopic exams with ocular dilation. Recommendations include providing comprehensive pre-session information on dilation risks, ensuring comfort during the exam, involving expert instructors, and addressing logistical concerns such as transportation. While this study provides useful guidelines, further research with a larger and more diverse sample of SPs is needed to refine these practices and better understand their experiences in similar educational settings.

## Figures and Tables

**Figure 1:**
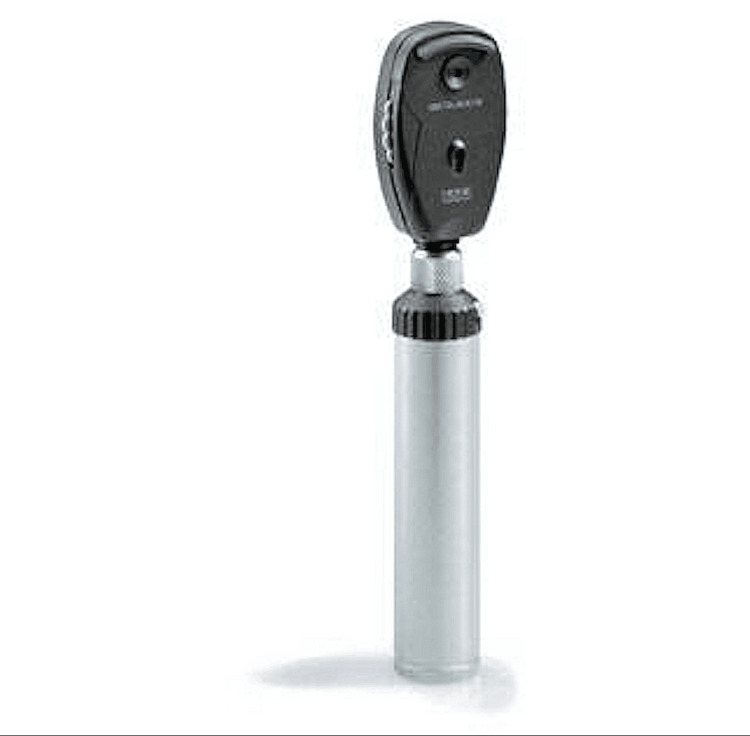
Direct ophthalmoscope

## Appendices

Appendix A: Standardized Patient Fundoscopy Pre-survey

Please enter a unique code that will allow us to anonymously link your responses to future surveys on this topic (ex, father's first initial, favorite number, birthday). Please write down or remember this code, as you will need it for the post survey! 

________________________________________________________________

What is your age?

________________________________________________________________

How long have you been a standardized patient (SP)?

o   (1)

o 1-3 years  (2)

o 3-5 years  (3)

o >5 years  (4)

How do you identify yourself?

o Male  (1)

o Female  (2)

o Non-binary  (3)

o Other   (4)

In what roles have you served as an SP? Please click all that apply.

▢        Interview encounters (in person)  (1)

▢        Interview encounters (virtual telehealth)  (2)

▢        Physical exam - heart exam  (3)

▢        Physical exam - lung exam  (4)

▢        Physical exam - abdominal exam  (5)

▢        Physical exam - head and neck exam  (6)

The next question refers to the image below: 

Have you been an SP for the "fundoscopic" exam (exam during which a tool with a light is shined into the eye to look into the back of the eye - see image above?)

o Yes  (1)

o No  (2)

If yes to the previous question, have you had eye drops which dilate the pupils as part of the fundoscopic exam?

o Yes  (1)

o No  (2)

What concerns (if any) do you have with serving as an SP for the fundoscopic exam (examination of the back of the eye with the tool shown in the previous image)?

________________________________________________________________

________________________________________________________________

What concerns (if any) do you have with receiving the eye drops that dilate the pupils?

________________________________________________________________

________________________________________________________________

What information would help alleviate these concerns?

________________________________________________________________

________________________________________________________________

Have you had an eye exam in the past?

o Yes  (1)

o No  (2)

If yes, were your eyes dilated during that exam?

o Yes  (1)

o No  (2)

When was your most recent eye exam?

▢        in the past year  (1)

▢        in the past 2-3 years  (2)

▢        in the past 5 years  (3)

▢        in the past 10 years  (4)

▢        Other   (5) __________________________________________________

What benefits do you see (if any) as serving as an SP for fundoscopy?

________________________________________________________________

________________________________________________________________

Appendix B: Standardized Patient Fundoscopy Post-survey

Please re-enter the unique code you used on the pre-survey which will allow us to anonymously link your survey responses (ex: father's first initial, favorite number, birthday)

________________________________________________________________

What is your age?

________________________________________________________________

How do you identify yourself?

o Male  (1)

o Female  (2)

o Non-binary  (3)

o Other   (4)

How was your experience serving as SP for the "fundoscopic" eye exam?

________________________________________________________________

________________________________________________________________

Did you receive eye drops for dilation?

o Yes in ONE eye  (1)

o Yes in BOTH eyes  (2)

o I did NOT receive any eye drops  (3)

o Other:  (4) __________________________________________________

How satisfied were you with the information about the dilation process

o Very unsatisfied  (1)

o Unsatisfied  (2)

o Neutral  (3)

o Satisfied  (4)

o Very satisfied  (5)

How satisfied were you with the information you received regarding what to expect from a student practicing the fundoscopic exam

o Very unsatisfied  (1)

o Unsatisfied  (2)

o Neutral  (3)

o Satisfied  (4)

o Very satisfied  (5)

Is there any additional information you feel would have been helpful to receive prior to the session?

________________________________________________________________

________________________________________________________________

How comfortable were you during the fundoscopic eye exam

o Very uncomfortable  (1)

o Uncomfortable  (2)

o Neutral  (3)

o Comfortable  (4)

o Very comfortable  (5)

What (if anything) would have made you more comfortable?

________________________________________________________________

________________________________________________________________

If you've served as an SP before, how did this experience compare to serving as an SP for other sessions (ie, interview sessions, physical exam sessions, etc)?

________________________________________________________________

________________________________________________________________

Is there any additional advice or information you would give to actors who will be serving as an SP for fundoscopy sessions?

________________________________________________________________

________________________________________________________________

Would you participate as an SP for fundoscopy again?

o Yes  (1)

o No  (2)

Would you participate as an SP with dilation again?

o Yes  (1)

o No  (2)

Were you asked to provide feedback to the students?

o Yes  (1)

o No  (2)

If you were asked to provide feedback to the students, what were some of the common comments you suggested?

________________________________________________________________

________________________________________________________________

If you were not asked to provide feedback to the students, did you still provide any informal feedback, and what comments did you make?

________________________________________________________________

________________________________________________________________
